# Genome-Wide Identification of Candidate Genes Underlying Soluble Sugar Content in Vegetable Soybean (*Glycine max* L.) *via* Association and Expression Analysis

**DOI:** 10.3389/fpls.2022.930639

**Published:** 2022-08-04

**Authors:** Wencheng Lu, Meinan Sui, Xunchao Zhao, Hongchang Jia, Dezhi Han, Xiaofei Yan, Yingpeng Han

**Affiliations:** ^1^Heihe Branch of Heilongjiang Academy of Agricultural Sciences, Heihe, China; ^2^Key Laboratory of Soybean Biology in Chinese Ministry of Education (Key Laboratory of Soybean Biology and Breeding/Genetics of Chinese Agriculture Ministry), Northeast Agricultural University, Harbin, China

**Keywords:** genome-wide association analysis, candidate genes, soluble sugar, vegetable soybean, single nucleotide polymorphism

## Abstract

Soluble sugar is a major indicator of the intrinsic quality of vegetable soybean [*Glycine max* (L.) Merr. ]. The improvement of soluble sugar content in soybean is very important due to its healthcare functions for humans. The genetic mechanism of soluble sugar in soybean is unclear. In this study, 278 diverse soybean accessions were utilized to identify the quantitative trait nucleotides (QTNs) for total soluble sugar content in soybean seeds based on a genome-wide association study (GWAS). A total of 25,921 single-nucleotide polymorphisms (SNPs) with minor allele frequencies (MAFs) ≥ 5% and missing data ≤ 10% were selected for GWAS. Totally, thirteen QTNs associated with total soluble sugar content were identified, which were distributed on ten chromosomes. One hundred and fifteen genes near the 200-kb flanking region of these identified QTNs were considered candidate genes associated with total soluble sugar content in soybean seed. Gene-based association analysis and haplotype analysis were utilized to further identify the effect of candidate genes on total soluble sugar content. Totally, 84 SNPs from seventeen genes across four chromosomes were significantly associated with the total soluble sugar content. Among them, three SNPs from *Glyma.02G292900* were identified at two locations, and other eighty-one SNPs from sixteen genes were detected at three locations. Furthermore, expression level analysis of candidate genes revealed that *Glyma.02G293200* and *Glyma.02G294900* were significantly positively associated with soluble sugar content and *Glyma.02G294000* was significantly negatively associated with soluble sugar content. Six genes (i.e., *Glyma.02G292600, Glyma.02G292700, Glyma.02G294000, Glyma.02G294300, Glyma.02G294400*, and *Glyma.15G264200*) identified by GWAS were also detected by the analysis of differential expression genes based on soybean germplasms with higher and lower soluble sugar content. Among them, *Glyma.02G294000* is the only gene that was identified by gene-based association analysis with total soluble sugar content and was considered an important candidate gene for soluble sugar content. These candidate genes and beneficial alleles would be useful for improving the soluble sugar content of soybean.

## Introduction

The soybean [*Glycine max* (L.) Merr.] seeds are widely utilized in the processing of soybean products, such as soy milk, tofu, and bean paste. Approximately, 40 g protein, 35 g carbohydrates, 20 g oil, and 5 g ash are present in 100 g of dried soybean seeds (Krober and Cartter, 1962). Among carbohydrates, the concentration of soluble sugars is as high as 16.6% (Hymowitz et al., 1974), which contributes to the quality and nutritional values of soy food. The primary component of total soluble sugar in mature soybean seeds is sucrose, accounting for about 5% of soybean dry matter, and its improvement is beneficial for the taste and sweetness of soybean products. The other two components that play an important role are raffinose and stachyose, accounting for 1.5 and 3% of the total soluble sugar content in soybean dry matter, respectively (Hymowitz et al., 1972; Openshaw and Hadley, 1978; Kuo et al., 1988; Wilson, 2004), which have a great influence on the fabric of soybean fresh food and soybean products. There are also other minor sugars in soybean seeds, such as glucose, fructose, and galactose, which account for a small proportion of soluble carbohydrates, <1% totally (Hymowitz et al., 1972). Thus, improving soluble sugar contents in soybean, such as increasing sucrose content and reducing raffinose and stachyose contents, plays a vital role in soybean genetic breeding and quality improvement, which has attracted the attention of soybean breeders.

Vegetable soybeans (*G. max* (L.) Merr.) are also known as edamame, which is harvested as a green and fully filled pod when the seeds are ~80% mature. Vegetable soybeans are rich in protein, soluble sugars, starch, dietary fiber, minerals, vitamins, and other phytochemicals, such as isoflavones, with anticancer and other health-promoting activities (Kumar et al., 2011; Kao et al., 2020). Due to the many nutritional benefits to humans, the demand for vegetable soybeans is likely to continue to increase in the future (Jiang et al., 2018). Sweetness, aroma, taste, and texture are important aspects of the edible quality of vegetable soybeans. Chemical substances that affect sweetness, taste, and flavor include sucrose, free amino acids, organic acids, inorganic salts, and flavonoids. Among them, the sweetness mainly depends on the content of sucrose, the texture and flavor depend on the content of free amino acids, and the content of lipoxygenase and hardness determines the beany flavor and softness of the mouthfeel. It is the most important and sustainable goal of vegetable soybean breeding to improve the overall quality of vegetable soybeans, such as appearance quality, nutritional quality, and taste quality, and increase the yield of vegetable soybeans (Golicz et al., 2016; Varshney et al., 2020).

The increasingly mature molecular marker technology is beneficial in soybean breeding. Currently, hundreds of quantitative trait loci (QTLs) related to agronomic and quality traits have been mapped in different populations across nearly all chromosomes of soybean, while only a small number of studies about a genetic study and QTL analysis of soluble sugars in soybean seeds were reported. Few QTLs related to soluble sugars (e.g., sucrose, raffinose, and stachyose) were detected, indicating that this field is worth considering. Kim et al. (2005) identified two QTLs for oligosaccharide and sucrose. Kim et al. (2006) detected two QTLs, located on Chr.12 and Chr.16, indicating that the phenotypic variation of sucrose content is lower than 10%. Maroof and Buss (2011) detected a major QTL on chromosome 11 (LG B1) for sucrose and stachyose contents. One QTL for sucrose and raffinose, and two QTLs for stachyose were detected in a set of 170 F_2:3_ RILs by Wang et al. (2014). Akond et al. (2015) identified fourteen significant QTLs associated with soluble sugar contents by an F_5:7_ population with 92 lines, including QTLs related to sucrose (three QTLs across three LGs), raffinose (seven QTLs across six LGs), and stachyose (four QTLs across four LGs) contents. However, due to the low resolution and density of the detected QTL, it is difficult to detect the QTLs related to soluble sugar content by molecular marker-assisted selection (MAS).

A genome-wide association analysis (GWAS) is thought to be a valid alternative to linkage analysis, its resolution and accuracy are higher than linkage analysis, and it plays a significant role in analyzing the genetic basis of soybean complex traits (Li et al., 2015). At present, GWAS has been widely utilized in genetic analysis of soybean quality breeding, for instance, protein content (Hwang et al., 2014), oil content (Hwang et al., 2014), fatty acid content (Cao et al., 2017), and amino acid content (Grant et al., 2010). The restricted two-stage multi-locus GWAS (RTM-GWAS) was considered a procedure that can comprehensively and effectively detect QTL, which has been applied widely nowadays (Khan et al., 2019). However, up to date, no studies related to the analysis of soluble sugar content in soybean seeds on the basis of genome sequencing technology had been reported.

In this study, 278 tested soybean germplasms and 25,921 polymorphic single-nucleotide polymorphism (SNP) markers were conducted for a GWAS of total soluble sugar content in fully developed grains of soybean. The purpose was to clarify the genetic structure of soluble sugar content in soybean grains and to unearth the candidate genes related to soluble sugar content by means of peak SNPs in soybean seeds, and the genes related to soybean soluble sugar content were screened and predicted by candidate gene-based association analysis and gene expression analysis, in order to provide a basis for the cultivation of soybean germplasm with a higher soluble sugar content.

## Materials and Methods

### Planting and Phenotyping

A panel of 278 soybean germplasms (landraces and elite cultivars) was collected to analyze the phenotypic variation and genotyping by sequencing (Supplementary Table S1). We planted the germplasms at three locations, including Harbin (117°17′E, 33°18′N), Gongzhuling (124°82′E, 43°50′N), and Shenyang (41°48′N, 123°25′E), in 2019. Three replications and a randomized complete block design were applied for field experimental trials. In addition, the single row plots of 3 m long and 0.65 m between rows were exploited in each tested environment. When plants reached full development, 10 randomly selected plants from each row in each plot were picked up to measure the total soluble sugar content.

### Measurement of Total Soluble Sugar Content

Total soluble sugar content in soybean seeds was extracted and quantified according to the method described by Taira (1990) and Fox and Robyt (1991). Approximately, 10 g of tested and fully developed grains of soybean were milled to a fine powder and dried. Then, 3 g sample, 200 ml distilled water, and 20 ml HCl [25% (w/w)] were mixed in a glass bottle (250 ml). The bottle was homogenized for 5 s by shaking and placed in a water bath at 100°C for 4 h. Later, the bottle was taken out and quickly cooled to room temperature in ice water for 20 min. A volume of 5.5 ml NaOH [40% (w/v)] was poured into the bottle, and then the mixture was filtered through a filter paper into a 500-ml glass filter flask after mixing the liquid upside down. The solution was transferred to a 500-ml volumetric flask and filled to calibration with distilled water and mix it. A volume of 3 ml solution was pipetted into a 100-ml volumetric flask and filled to calibration with distilled water to dilute to measurable concentration. After mixing, the modified phenol-sulfuric acid method was used for measuring the total soluble sugar content. A volume of 25 μl of each sample extract and 25 μl of 5% (w/v) phenol were placed in each well of a 96-well general assay plate; 125 μl of concentrated H_2_SO_4_ was poured into each well after placing the plate on the ice and mixing it for 30 s at a slow speed. The plate was sealed and then placed in a water bath at 80°C for 30 min. The absorbance at 490 nm was read in a Titertek Multiskan Plus spectrophotometer equipped for reading. The concentration of total sugar in each sample was determined by the standard curve. Each sample analysis was performed in triplicate.

### Genotyping Data

The genomic DNA of each sample from young leaf was isolated by the method of CTAB and sequenced *via* the specific locus amplified fragment sequencing (SLAF-seq) methodology (Sun et al., 2013). Two digest enzymes, *Mse*I (EC 3.1.21.4) and *Hae*III (EC: 3.1.21.4) (Thermo Fisher Scientific Inc., Waltham, MA, USA), were used to acquire more than 50,000 sequencing tags from each accession, and the length of the tags varied from 300 to 500 bp. The acquired sequencing tags of each accession, distributed in unique genomic regions of the twenty chromosomes in soybean, were used to define sequencing libraries. The alignment between the raw paired-end reads and soybean reference genome Williams 82 (version: Glyma.Wm82.a2) was performed *via* the Short Oligonucleotide Alignment Program 2 (SOAP2) software. The raw reads in the same genomic position were utilized to define the SLAF groups using more than 58,000 high-quality SLAF tags from each tested sample. When MAF ≥ 0.05, the SNPs were defined. As long as the depth of minor allele/the total depth of the sample is more than one-third of the total, then the genotype was defined to be heterozygous (Han et al., 2016).

### Population Structure Evaluation and Linkage Disequilibrium Analysis

The assessment approach for the population structure of the association panel was principle component analysis (PCA) of the GAPIT software (Lipka et al., 2012). The linkage disequilibrium (LD) block between pairs of SNPs (MAF ≥ 0.05 and missing data ≤ 10%) and *r*^2^ (squared allele frequency correlations) was calculated using TASSEL 3.0 (Bradbury et al., 2007). In contrast to the GWAS, missing SNP genotypes were not imputed with the major allele before LD analysis. The parameters in the program included MAF (≥0.05) and the integrity of each SNP (≥80%).

### Association Analysis

The RTM-GWAS procedure was performed to detect the signals associated with the total soluble sugar content in soybean seeds based on 25,921 SNPs and 278 soybean germplasms under three environments (He et al., 2017). Of this association study, two stages were conducted. The first stage was to preselect markers by a single-locus association test based on the simple linear model. The second stage was to detect genome-wide QTLs for preselected markers under the multi-locus multi-allele model featured with forward selection and backward elimination. In these two stages, the three eigenvectors with the largest eigenvalues of the GSC matrix calculated from the genome-wide SNPs were incorporated as covariates for population structure correction. The significance level used for preselection markers was *P* ≤ 0.05. In addition, the stepwise regression level was *P* ≤ 0.01. The conservative Bonferroni criterion was utilized here (Khan et al., 2019). The genotype-environment interaction also was considered.

### Prediction of Candidate Genes for Total Soluble Sugar Content

The 200-kb genomic region of each peak SNP was regarded as the candidate interval, and genes located in the interval were treated as candidate genes and then identified and annotated the candidate genes with the soybean reference genome (Wm82. a2. v1, http://www.soybase.org) (Cheng et al., 2017).

The SNP variations happened in a genomic region of candidate genes, including exon regions, splicing sites, untranslated regions (UTRs), intronic regions, and upstream and downstream regions, which were detected in twenty soybean lines with higher and lower soluble sugar content based on genome re-sequencing data. SNPs located in these regions of candidate genes with MAF <0.05 were filtered out, and then LD KNNI imputation was used to impute the genotypes of these SNPs. Association analysis was conducted by obtaining SNP data and total soluble sugar content using the general linear model (GLM) method in TASSEL version 5.0 for identifying total soluble sugar-related haplotypes (Bradbury et al., 2007). Significant SNPs affecting the target trait were asserted when the test statistics reached *P* < 0.01.

Candidate genes were further analyzed by analyzing the expression levels of 14 soybean germplasms, including seven germplasms, each with high and low-soluble sugar content. RNA purification kit (TIANGEN, DP432) was used to extract total RNA from soybean seeds. Then, first-strand cDNA was synthesized according to the TIANScript RT kit (TIANGEN, KR104). Real-time fluorescence quantification was conducted by ABI 7500 Fast using SuperRealPreMix Plus (SYBR Green) Kit (TIANGEN, FP205) to confirm the transcript abundance of candidate genes. The reaction systems for reverse transcription and real-time fluorescence quantification were performed according to the manufacturer's instructions. The real-time PCR programs were as follows: holding stage: 95°C for 30 s; cycling stage: 95°C for 3 s, 60°C for 30 s, and 72°C for 30 s for 40 cycles; melt curve stage: 95°C for 15 s, 60°C for 1 min, 95°C for 30 s, and 60°C for 15 s. Three technical replicates and three biological duplications were conducted for each sample. A comparative threshold method (2^−ΔΔCT^) was utilized to calculate the relative transcript levels of candidate genes in diverse soybean germplasm. *GmActin4* (GenBank accession no. AF049106) was considered the internal standard control. The primer sequences for real-time fluorescence quantitative analysis are shown in Supplementary Table S2.

### RNA Sequencing and Co-Expression Analysis

According to results from the 278 tested soybean varieties, seven lines with high-soluble sugar content and seven lines with low-soluble sugar content were chosen for RNA-seq analysis. Seeds were collected at the fully developed stage, and each sample had two replicates. The collected samples were rapidly put into liquid nitrogen for freezing and stored at −80°C. Total RNA was extracted from twenty-eight soybean lines using TRIzol reagent (Invitrogen). The quality of RNA samples was examined, and then libraries were successfully constructed. All constructed libraries were sequenced using the Illumina platform.

The differential gene expression (DEG; |log2FC| ≥1.5) was used to calculate the Pearson correlation coefficient. Self-matches and duplicates were removed, and the Pearson correlation cutoff value of 0.87 was obtained. The achieving and visualizing of the network were applied using the Cytoscape 3.6.0 software.

## Results

### Statistical and Variation Analysis of Total Soluble Sugar Content

**Figure 1:**
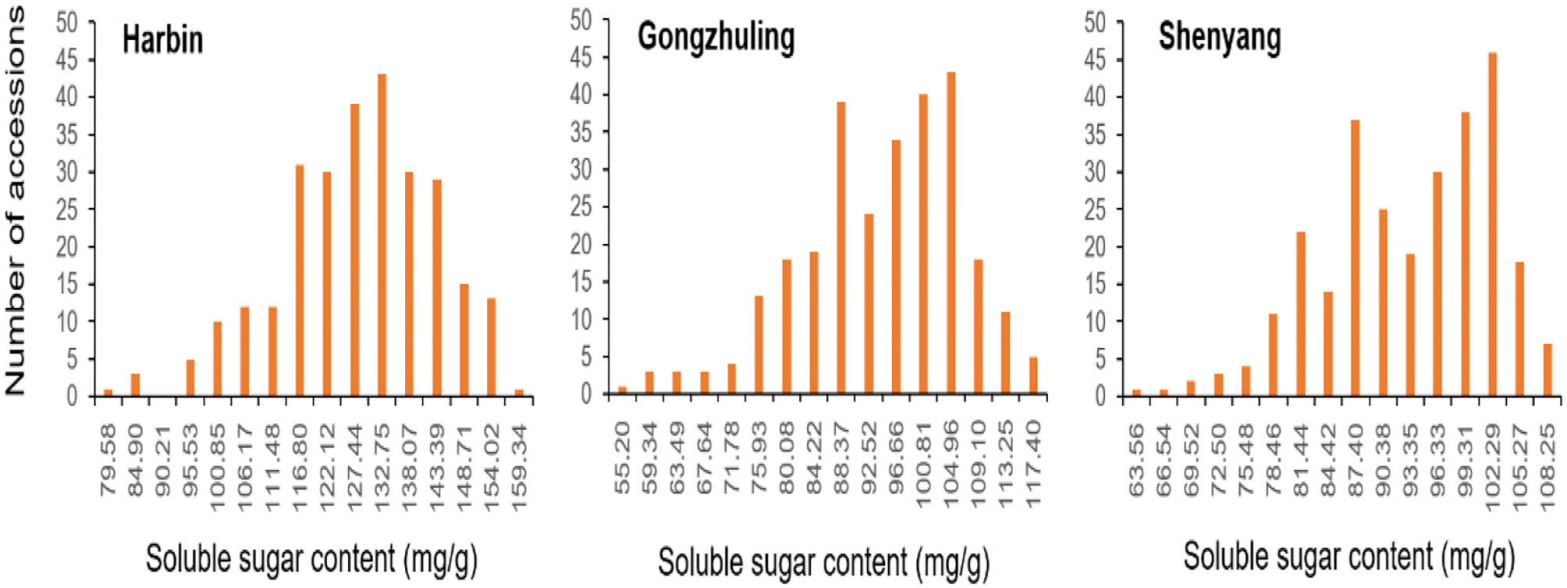
Variation of total soluble sugar content among 278 accessions in three environments (“Harbin,” “Gongzhuling,” and “Shenyang”).

As shown in Table 1 and Figure 1, the total soluble sugar content showed a wide range of variation in three test environments on the basis of 278 soybean germplasms, for instance, minimum, maximum, means, coefficient of variation (CV%), skewness, and kurtosis. The phenotypic value varied a lot among different locations, especially the phenotypic value range of “Harbin” (from 79.58 to 164.66 mg/g) was higher than that of “Gongzhuling” (from 55.20 to 121.53 mg/g) and “Shenyang” (from 63.56 to 111.22 mg/g). The CVs% under three locations were observed from 10.52 to 12.89%. Simultaneously, the kurtosis and skewness of the association panel we observed in all tested environments were normal and without any saliency, which indicated that it was suitable for GWAS analysis.

**Table 1:** Statistical analysis and variation of total soluble sugar content of association panel.

**Location**	**Minimum**	**Maximum**	**Mean**	**Coefficient of variation (%)**	**Skewness**	**Kurtosis**
Harbin	79.58	164.66	126.02	12.44	−0.27	0.21
Gongzhuling	55.20	121.53	92.37	10.52	−0.43	−0.29
Shenyang	63.56	111.22	93.23	12.89	−0.43	0.11

### Sequencing and Genotyping

**Figure 2:**
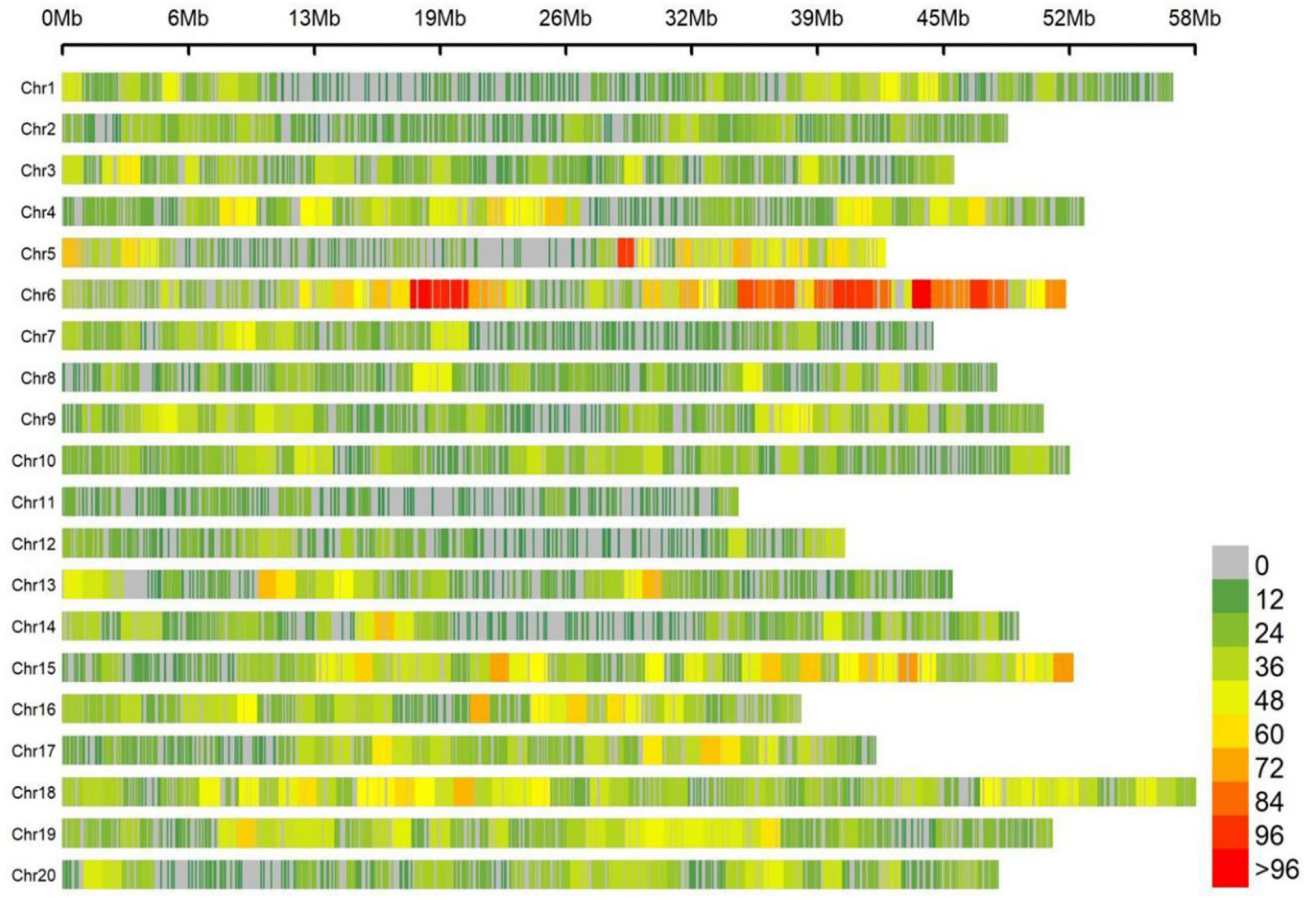
Single-nucleotide polymorphism (SNP) density and distribution across 20 soybean chromosomes.

Totally, 25,921 high-quality markers (MAF ≥ 0.05, missing data ≤ 10%) from the genomic DNA of an association panel consisting of 278 soybean germplasms were utilized for genotyping in view of the SLAF-seq approach. These SNPs spanned a wide range of the twenty chromosomes of soybean genome, with a range of 946.7 Mbp totally, accounting for ~86.06% of the soybean genome (Figure 2). Diversity of marker density were observed among different chromosomes with an average distance of 36.52 kb between two SNPs and an average number of 1,296 SNPs per chromosome, which varied from the largest number of 2,894 SNPs in Chr.06 to the fewest number of 385 SNPs in Chr.11.

### Analysis of Linkage Disequilibrium and Population Structure

**Figure 3:**
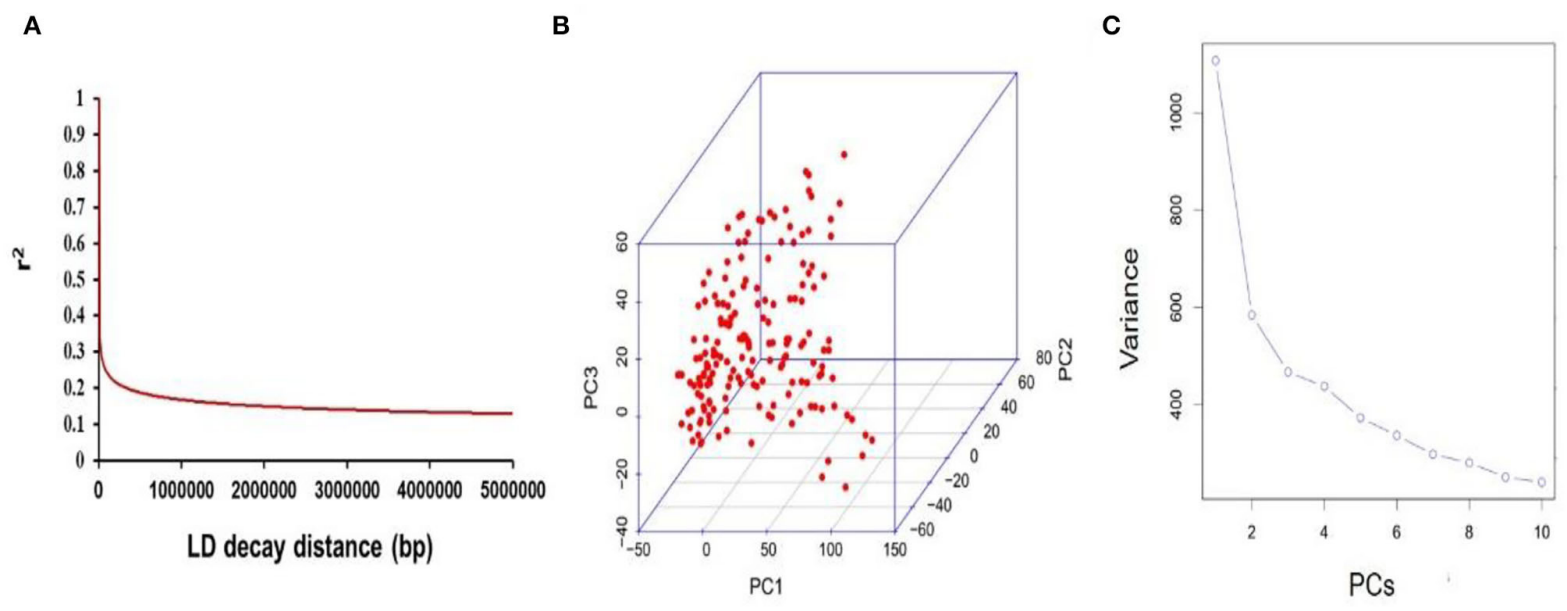
Mapping genetic data of populations. **(A)** Linkage disequilibrium (LD) decay of genome-wide association study (GWAS) population. **(B)** The first three PCs of 25,921 SNPs used in the GWAS. **(C)** Population structure of soybean germplasms.

The mapping resolution for genome scans and GWA mapping were depicted by analyzing the average extent of LD decay distance. The mean LD decay distance of the panel was ~216.13 kb, when *r*^2^ drops to 0.4 (Figure 3A). A total of 25,921 SNPs were used for scanning the population stratification of association panels through the principal component (PC) and kinship analysis. The results demonstrated that the first three PCs accounted for 14.94% of the genetic variation, PC2 was the point that the entire genetic variation sharply declined, and PC3 was the inflection point, which demonstrated that the first three PCs uncommonly affect the mapping population (Figures 3B,C).

### Quantitative Trait Nucleotides Associated With Total Soluble Sugar Content by GWAS

**Figure 4:**
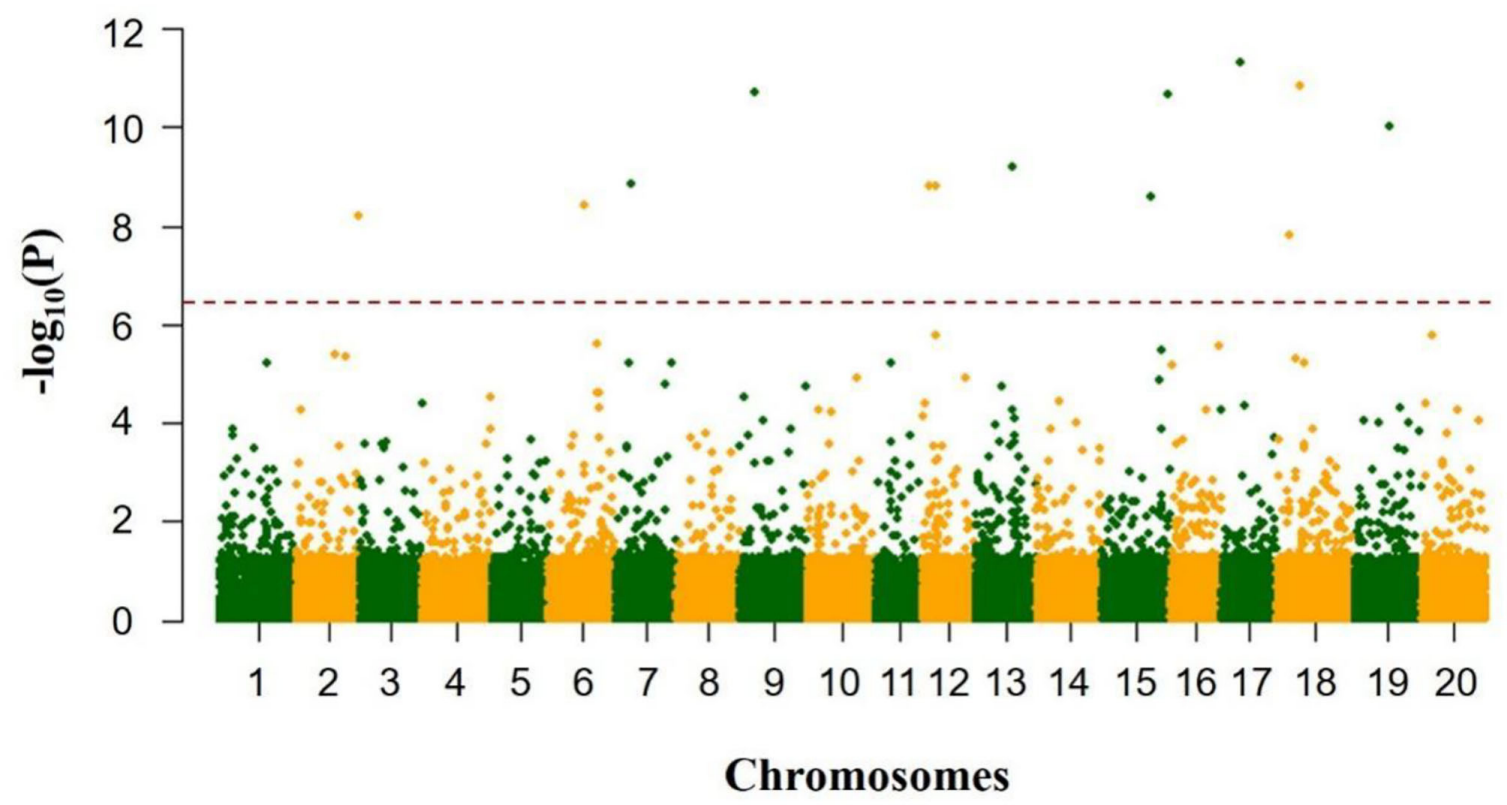
GWAS results of total soluble sugar content from 278 soybean accessions.

Totally, thirteen quantitative trait nucleotides (QTNs) that covered ten chromosomes associated with the total soluble sugar content were determined based on RTM-GWAS (Table 2; Figure 4), including rs47259089 on Chr.02, rs27824573 on Chr.06, rs10931728 on Chr.07, rs11039122 on Chr.09, rs5036567 and rs10157456 on Chr.12, rs28054318 on Chr.13, rs36090182 and rs49892059 on Chr.15, rs14145078 on Chr.17, rs9733379 and rs17293362 on Chr.18, and rs25527752 on Chr.19. To confirm whether these detected QTNs are related to total soluble sugar content, the effects of alleles were evaluated. By comparison, the total soluble sugar content of soybean germplasm containing different alleles was found, which indicated that the difference in alleles significantly affected the total soluble sugar content (Table 2). Hence, these appropriate alleles would be beneficial for MAS of soybean with suitable soluble sugar content.

**Table 2:** Peak SNP and beneficial allele associated with total soluble sugar content identified by GWAS.

**SNP**	**Chr**.	**Position**	**–log10(P)**	**Location**	**Allele 1**	**Allele 2**	**Average soluble sugar concentration of accessions with allele 1**	**Average soluble sugar concentration of accessions with allele 2**	**Average soluble sugar concentration of population**
rs47259089	2	47259089	8.20	Gongzhuling	A	C	93.81	89.34	92.37
				Harbin			128.03	121.76	126.02
				Shenyang			94.82	89.88	93.23
rs27824573	6	27824573	8.43	Gongzhuling	A	C	93.17	85.96	92.37
				Harbin			127.27	115.31	126.02
				Shenyang			94.21	84.74	93.23
rs10931728	7	10931728	8.85	Gongzhuling	A	C	92.85	91.06	92.37
				Harbin			126.71	124.11	126.02
				Shenyang			93.77	91.74	93.23
rs11039122	9	11039122	10.73	Gongzhuling	T	C	99.70	91.94	92.37
				Harbin			138.00	125.31	126.02
				Shenyang			102.42	92.69	93.23
rs5036567	12	5036567	8.82	Gongzhuling	T	G	94.08	80.49	92.37
				Harbin			127.10	107.69	126.02
				Shenyang			93.08	78.94	93.23
rs10157456	12	10157456	8.80	Gongzhuling	T	G	95.17	91.68	92.37
				Harbin			131.08	124.81	126.02
				Shenyang			96.91	92.33	93.23
rs28054318	13	28054318	9.19	Gongzhuling	A	T	96.11	92.08	92.37
				Harbin			133.29	125.43	126.02
				Shenyang			98.40	92.81	93.23
rs36090182	15	36090182	8.58	Gongzhuling	T	A	97.26	91.57	92.37
				Harbin			133.53	124.88	126.02
				Shenyang			99.12	92.36	93.23
rs49892059	15	49892059	10.69	Gongzhuling	T	C	97.83	91.52	92.37
				Harbin			134.88	124.64	126.02
				Shenyang			100.21	92.14	93.23
rs14145078	17	14145078	11.32	Gongzhuling	C	T	94.24	83.18	92.37
				Harbin			128.94	111.60	126.02
				Shenyang			95.51	81.98	93.23
rs9733379	18	9733379	7.79	Gongzhuling	A	C	93.40	89.55	92.37
				Harbin			127.79	121.12	126.02
				Shenyang			94.56	89.55	93.23
rs17293362	18	17293362	10.85	Gongzhuling	T	C	94.27	89.00	92.37
				Harbin			129.07	120.52	126.02
				Shenyang			95.61	88.96	93.23
rs25527752	19	25527752	10.02	Gongzhuling	T	C	93.71	88.63	92.37
				Harbin			128.11	120.28	126.02
				Shenyang			94.89	88.61	93.23

### Prediction of Candidate Genes Controlling Total Soluble Sugar Content

The 200-kb flanking regions of the thirteen identified QTNs were thought to be candidate regions, and a total of 115 genes with functional annotation were in regions including four genes with unknown functional annotation (Table 3). A total of 107 genes among them were related to cell, cell wall, co-factor and vitamin metabolism, development, DNA, hormone metabolism, lipid metabolism, misc, nucleotide metabolism, protein, PS, redox, RNA, secondary metabolism, signaling, Tricarboxylic Acid/organic transformation, stress, and transport, and other eight genes were not assigned (Supplementary Figure S1).

**Table 3:** Gene models in the flanking regions of peak SNPs.

**Peak SNP**	**Chr**.	**Physical position (bp)**	**Gene model**	**Start position**	**Stop position**	**Functional annotation**
rs47259089	2	47259089	Glyma.02G292600	47161459	47162622	B-cell receptor-associated 31-like
			Glyma.02G292700	47162761	47163594	Stigma-specific Stig1 family protein
			Glyma.02G292800	47164820	47167693	Mannose-1-phosphate Guanylyltransferase (GDP)s;GDP-galactose:mannose-1-phosphate Guanylyltransferases;GDP-galactose:glucose-1-phosphate Guanylyltransferases;GDP-galactose:myoinositol-1-phosphate Guanylyltransferases;glucose-1-phosphate guanylyltransferase
			Glyma.02G292900	47175297	47278910	Transcription factor IIIA
			Glyma.02G293100	47183443	47188007	Cell differentiation, Rcd1-like protein
			Glyma.02G293200	47193354	47198311	SWAP (Suppressor-of-White-APricot)/surp domain-containing protein
			Glyma.02G293300	47200116	47202645	Transcription factor IIIA
			Glyma.02G293400	47204183	47206522	WRKY family transcription factor
			Glyma.02G293500	47207122	47210421	PYRIMIDINE B
			Glyma.02G293600	47211961	47216578	Co-factor for nitrate, reductase and xanthine dehydrogenase 5
			Glyma.02G293900	47225431	47228705	Glycosyl hydrolase 9B8
			Glyma.02G294000	47237993	47240495	O-Glycosyl hydrolases family 17 protein
			Glyma.02G294100	47248664	47250309	Ethylene response factor 7
			Glyma.02G294200	47256480	47263517	EPS15 homology domain 1
			Glyma.02G294300	47265184	47266385	Domain of unknown function (DUF2431)
			Glyma.02G294400	47267552	47269384	Hydroxyproline-rich glycoprotein family protein
			Glyma.02G294500	47272683	47277831	Transketolase family protein
			Glyma.02G294600	47280930	47285905	Co-chaperone GrpE family protein
			Glyma.02G294700	47288753	47291014	Pectin lyase-like superfamily protein
			Glyma.02G294800	47296020	47298094	F-box/RNI-like superfamily protein
			Glyma.02G294900	47300149	47306133	Trigger factor type chaperone family protein
			Glyma.02G295000	47306160	47312036	Phosphatidate cytidylyltransferase family protein
			Glyma.02G295100	47319345	47323749	Microtubule-associated proteins 65-1
			Glyma.02G295200	47325496	47328300	Hydroxyproline-rich glycoprotein family protein
			Glyma.02G295300	47333361	47337899	IAA carboxylmethyltransferase 1
			Glyma.02G295400	47344189	47352046	RING/FYVE/PHD zinc finger superfamily protein
			Glyma.02G295500	47355792	47360519	Lipid-binding serum glycoprotein family protein
rs10931728	7	10931728	Glyma.07G109400	10870758	10871858	Matrixin family protein
			Glyma.07G109500	10927821	10921254	Squamosa promoter binding protein-like 9
rs11039122	9	11039122	Glyma.09G087100	11123368	11127463	OPC-8:0 CoA ligase1
			Glyma.09G087200	11137727	11145131	Inositol transporter 2
rs5036567	12	5036567	Glyma.12G067700	4960025	4964029	PDI-like 1-4
			Glyma.12G067800	4971285	4972459	Proline-rich family protein
			Glyma.12G067900	4981283	4983658	DHBP synthase RibB-like alpha/beta domain
			Glyma.12G068000	4986769	4992981	RING/U-box superfamily protein
			Glyma.12G068100	5005644	5011265	Zinc finger (CCCH-type) family protein
			Glyma.12G068200	5012944	5024934	F-box/RNI-like superfamily protein
			Glyma.12G068300	5026726	5029278	Galactose oxidase/kelch repeat superfamily protein
			Glyma.12G068500	5035420	5042698	Geminivirus rep interacting kinase 2
			Glyma.12G068700	5044999	5046917	Late embryogenesis abundant (LEA) protein-related
			Glyma.12G068800	5049538	5050971	Late embryogenesis abundant (LEA) protein-related
			Glyma.12G068900	5053020	5054133	FASCICLIN-like arabinogalactan-protein 11
			Glyma.12G069000	5055071	5060301	beta-galactosidase 8
			Glyma.12G069100	5064839	5067048	nuclear factor Y, subunit C10
			Glyma.12G069200	5077641	5078829	nuclear factor Y, subunit C10
			Glyma.12G069300	5081048	5082478	FASCICLIN-like arabinogalactan-protein 11
			Glyma.12G069400	5085850	5087029	FASCICLIN-like arabinogalactan-protein 11
			Glyma.12G069500	5089679	5090763	FASCICLIN-like arabinogalactan-protein 11
			Glyma.12G069700	5097113	5098394	FASCICLIN-like arabinogalactan-protein 11
			Glyma.12G069800	5105383	5106659	FASCICLIN-like arabinogalactan-protein 11
			Glyma.12G069900	5109716	5111033	FASCICLIN-like arabinogalactan-protein 11
			Glyma.12G070000	5113528	5114869	FASCICLIN-like arabinogalactan-protein 11
			Glyma.12G070100	5118010	5127337	binding
			Glyma.12G070200	5129941	5131534	FASCICLIN-like arabinogalactan-protein 11
rs10157456	12	10157456	Glyma.12G108900	10083509	10088743	Auxin-responsive GH3 family protein
			Glyma.12G109000	10201437	10202411	zinc ion binding;nucleic acid binding
rs28054318	13	28054318	Glyma.13G164800	27967025	27969167	PLATZ transcription factor family protein
			Glyma.13G164900	27975423	27977287	chlororespiratory reduction 6
			Glyma.13G165100	27991996	27994467	glutamate receptor 3.4
			Glyma.13G165200	27997373	27999343	ribosomal protein S11-beta
			Glyma.13G165300	28011615	28015224	DNA-binding protein phosphatase 1
			Glyma.13G165400	28016508	28021594	Ribosomal protein S5/Elongation factor G/III/V family protein
			Glyma.13G165500	28030788	28034710	RING/U-box superfamily protein
			Glyma.13G165700	28056810	28059907	Nucleoside transporter family protein
			Glyma.13G165800	28061619	28064257	Tetratricopeptide repeat (TPR)-like superfamily protein
			Glyma.13G166000	28071252	28073533	SU(VAR)3-9 homolog 4
			Glyma.13G166100	28076313	28083963	Protein kinase superfamily protein
			Glyma.13G166200	28093807	28097420	EIN3-binding F box protein 1
			Glyma.13G166400	28116880	28119189	Ribosomal protein L36
			Glyma.13G166600	28139579	28142969	Ypt/Rab-GAP domain of gyp1p superfamily protein
			Glyma.13G166700	28151956	28153572	Integrase-type DNA-binding superfamily protein
rs49892059	15	49892059	Glyma.15G264000	49812996	49814711	myb domain protein 67
			Glyma.15G264100	49820324	49824883	NAC domain containing protein 20
			Glyma.15G264200	49844076	49848069	GDSL-motif lipase 5
			Glyma.15G264300	49856796	49861158	GDSL-motif lipase 5
			Glyma.15G264400	49871650	49872488	Peroxidase superfamily protein
			Glyma.15G264500	49874077	49879514	Ribosomal protein L18e/L15 superfamily protein
			Glyma.15G264600	49881177	49881683	HSP20-like chaperones superfamily protein
			Glyma.15G264700	49887796	49888398	FAR1-related sequence 9
			Glyma.15G264900	49890636	49892438	Tetratricopeptide repeat (TPR)-like superfamily protein
			Glyma.15G265000	49899364	49901747	Tetratricopeptide repeat (TPR)-like superfamily protein
			Glyma.15G265100	49903499	49905856	FAR1-related sequence 5
			Glyma.15G265200	49930318	49933567	DNA glycosylase superfamily protein
			Glyma.15G265300	49956889	49993042	Nuclear pore anchor
rs14145078	17	14145078	Glyma.17G161500	14073346	14079310	Phototropic-responsive NPH3 family protein
			Glyma.17G161600	14084020	14097997	Phototropic-responsive NPH3 family protein
			Glyma.17G161700	14112331	14114364	Acyl-CoA N-acyltransferases (NAT) superfamily protein
			Glyma.17G161800	14135127	14144033	Protein kinase superfamily protein
			Glyma.17G161900	14147978	14153289	Acetamidase/Formamidase family protein
			Glyma.17G162000	14172324	14173731	LOB domain-containing protein 38
			Glyma.17G162100	14197866	14199879	myb domain protein 79
rs9733379	18	9733379	Glyma.18G094500	9644898	9649165	Zinc finger (C2H2 type) family protein
			Glyma.18G094600	9649250	9650174	Heat shock protein 21
			Glyma.18G094700	9650627	9659495	Tetratricopeptide repeat (TPR)-like superfamily protein
			Glyma.18G094900	9685585	9686437	Pyridoxal phosphate (PLP)-dependent transferases superfamily protein
			Glyma.18G095100	9704195	9707617	GDSL-like Lipase/Acylhydrolase superfamily protein
			Glyma.18G095200	9732437	9738098	DNA-binding bromodomain-containing protein
			Glyma.18G095300	9740941	9744176	Tetraacyldisaccharide 4\'-kinase family protein
			Glyma.18G095400	9748448	9755084	DNA-binding bromodomain-containing protein
			Glyma.18G095600	9810199	9814709	myb domain protein 101
			Glyma.18G095800	9820014	9821451	Protein of unknown function, DUF584
rs17293362	18	17293362	Glyma.18G127800	17211254	17211713	Transmembrane receptors;ATP binding
			Glyma.18G127900	17212882	17215294	Disease resistance protein (TIR-NBS class), putative
			Glyma.18G128000	17217146	17219879	Disease resistance protein (TIR-NBS-LRR class) family
			Glyma.18G128100	17235800	17237647	Tetratricopeptide repeat (TPR)-like superfamily protein
			Glyma.18G128200	17243569	17246715	Protein of unknown function (DUF569)
			Glyma.18G128300	17285439	17289805	Dentin sialophosphoprotein-related
			Glyma.18G128400	17290689	17300339	Glutathione S-transferase family protein
			Glyma.18G128500	17303442	17305139	Pentatricopeptide repeat (PPR-like) superfamily protein
			Glyma.18G128600	17334538	17338674	Galactosyltransferase family protein
			Glyma.18G128700	17344300	17345252	Leucine-rich repeat protein kinase family protein
			Glyma.18G128800	17355977	17359186	Protein of unknown function (DUF1336)
rs25527752	19	25527752	Glyma.19G072200	25481614	25482042	RHO guanyl-nucleotide exchange factor 3
			Glyma.19G072300	25487042	25487576	ADP-glucose pyrophosphorylase family protein
			Glyma.19G072400	25499079	25505567	Proteasome family protein

Among these genes, *Glyma.02G293900* (located 33.658 kb near Chr02: 47259089 on Chr.2), belonged to glycosyl hydrolase, was directly involved in the carbohydrate metabolic process, which could catalyze cellulose to form cellobiose. *Glyma.02G294000* (located 21.096 kb near Chr02: 47259089 on Chr.2), a member of O-Glycosyl hydrolases family 17 protein, was involved in the starch and sucrose metabolism, which catalyzes the synthesis of D-glucose. *Glyma.12G069000* (located 18.504 kb near Chr12: 5036567 on Chr.12) was a beta-galactosidase 8, and it plays a catalytic role in the process of galactose metabolism and promotes the formation of D-galactose. *Glyma.19G072300* (located 40.71 kb near Chr19: 25527752 on Chr.19) was an ADP-glucose pyrophosphorylase family protein, which was involved in the carbohydrate metabolic process.

**Figure 5:**
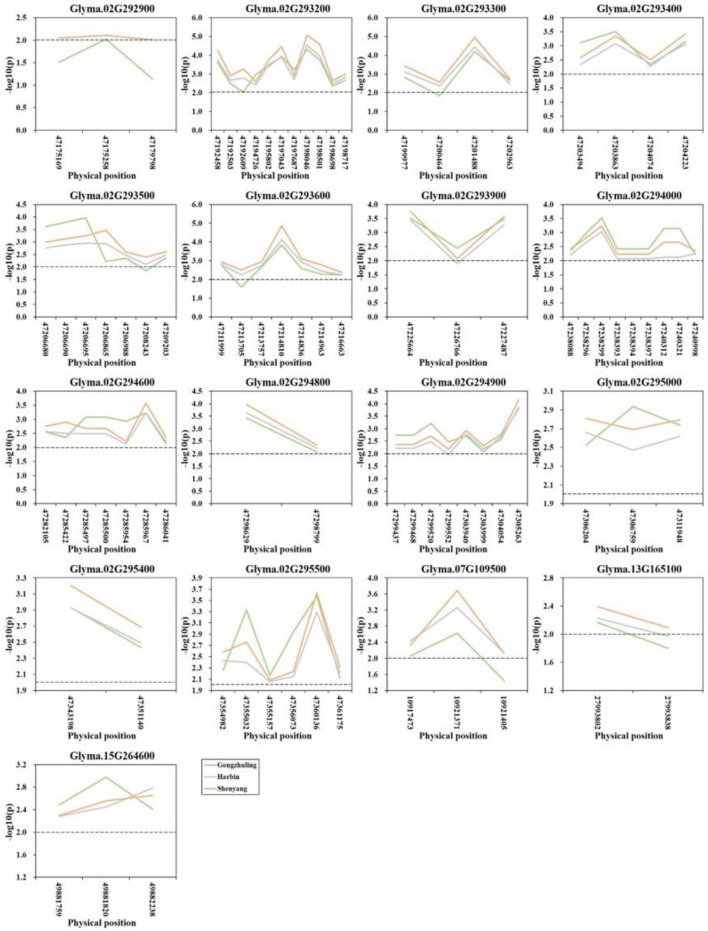
Candidate gene-based association analysis with total soluble sugar content. Horizontal line indicates that the threshold is set to 2.0.

A gene-based association analysis was performed based on the GLM method to predict the potential effects of candidate genes associated with total soluble sugar content. Through the genome resequencing of twenty lines, including ten higher and ten lower total soluble sugar lines, a total of 117 SNPs with MAF ≥ 0.05 were identified among thirty-two candidate genes. Totally, 84 SNPs from seventeen genes across four chromosomes were significantly associated with the total soluble sugar content. Among them, three SNPs from the gene *Glyma.02G292900* were detected at two environments, and other eighty-one SNPs from seventeen genes were detected at all three environments (Figure 5; Supplementary Table S3). Among them, *Glyma.02G293900* was reported to be involved in the carbohydrate metabolic process, which is useful for the synthesis of cellobiose, and may further affect sucrose content. *Glyma.02G294000* was a member of O-Glycosyl hydrolases family 17 protein, which was involved in the process of starch and sucrose metabolism, which may affect the degradation of UDP-glucose and then influence the soluble sugar content. Other genes could be regarded as novel genes affecting the total soluble sugar content of soybean. The allelic effects of different alleles between each significant SNP were analyzed, and the results demonstrated that there was a significant difference in the soluble sugar content of soybean germplasm under different alleles of twenty candidate genes (Supplementary Table S4). Thus, these beneficial alleles from candidate genes would be useful for MAS in soybeans with a higher soluble sugar content.

**Figure 6:**
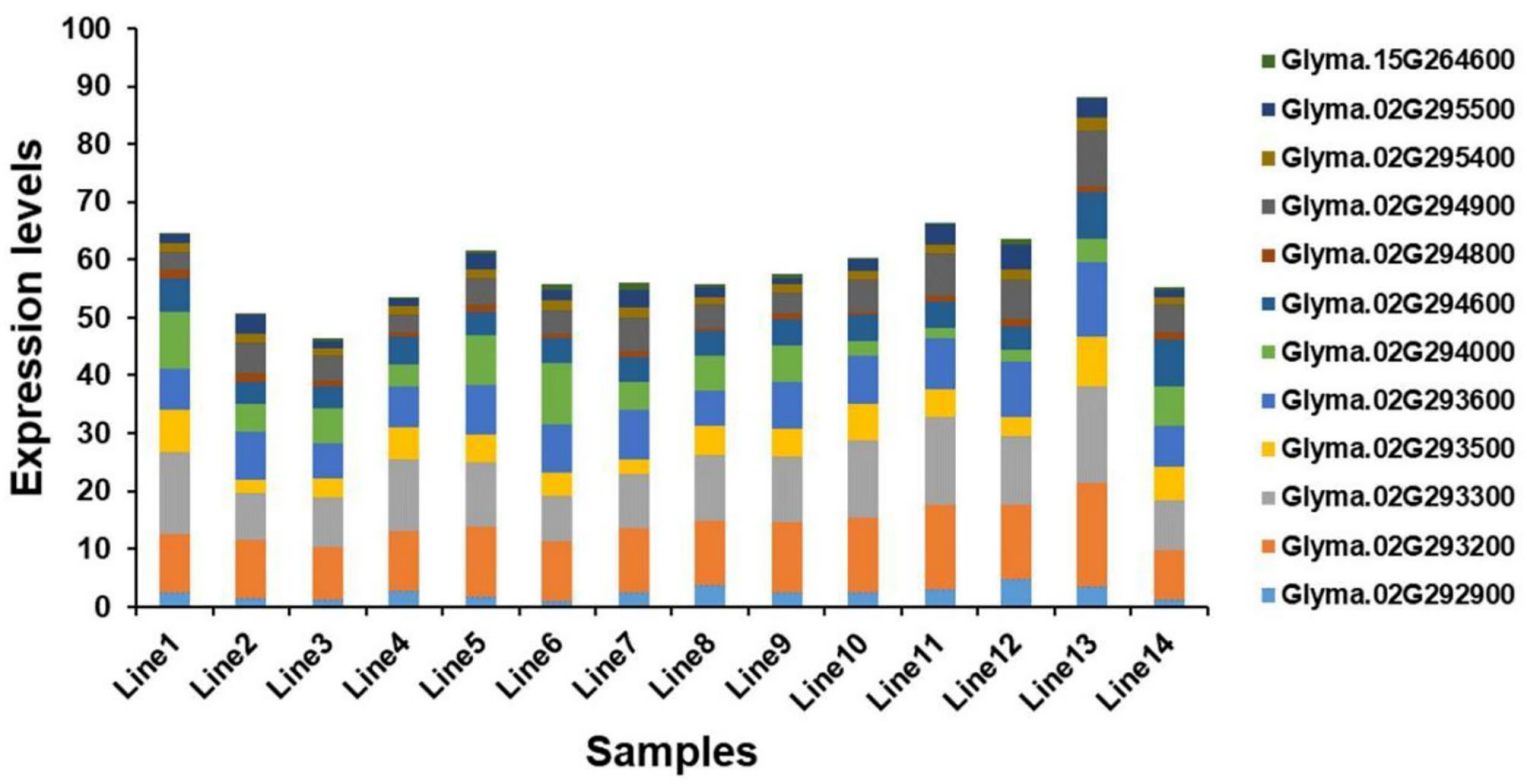
Expression level analysis of candidate genes. Line 1 to Line 7 are germplasms with low-soluble sugar content, and Line 8 to Line 14 are germplasms with high-soluble sugar content.

Seventeen candidate genes obtained by haplotype analysis were subjected to expression analysis to further dissect their potential functions, which was based on seven of each soybean germplasm with high- and low-soluble sugar content (Supplementary Figure S2). Among these genes, five genes (i.e., *Glyma.02G293400, Glyma.02G293900, Glyma.02G295000, Glyma.07G109500*, and *Glyma.13G165100*) showed low expression or almost no expression in soybean seeds, and the expression levels of other genes in different germplasms showed some differences. The expression levels of the two candidate genes (e.g., *Glyma.02G294000* and *Glyma.02G294800*) in the low-soluble sugar germplasm showed an overall slightly higher expression level than in the high-soluble sugar content germplasm, and other genes showed the opposite trend (Figure 6). We analyzed the correlation between the expression levels of these twelve candidate genes expressed in soybean seeds and soluble sugar content using Pearson's correlation coefficients. As a result, the expression levels of *Glyma.02G293200* and *Glyma.02G294900* showed a significant positive correlation with soluble sugar content. There was a significant negative correlation between the expression of *Glyma.02G294000* and soluble sugar content. The expression levels of other genes showed no significant correlation with soluble sugar content (Supplementary Table S5). These candidate genes detected may play a key role in the regulation of soluble sugar content in soybean seeds.

### Construction of Gene Co-Expression Networks Related to Soluble Sugar Content

The DEG (|log2FC| ≥1.5) was detected between soybean lines with high- and low-soluble sugar content and was used to construct the gene co-expression networks of candidate genes. A total of 1,291 DEGs were identified. Among the candidate genes underlying soluble sugar content of soybean seeds, six genes were commonly found with DEG and GWAS, including genes encoding a B-cell receptor-associated 31-like (*Glyma.02G292600*), Stigma-specific Stig1 family protein (*Glyma.02G292700*), O-Glycosyl hydrolases family (*Glyma.02G294000*), Domain of unknown function (*Glyma.02G294300*), hydroxyproline-rich glycoprotein family protein (*Glyma.02G294400*), and GDSL-motif lipase 5 (*Glyma.15G264200*) (Supplementary Table S6).

**Figure 7:**
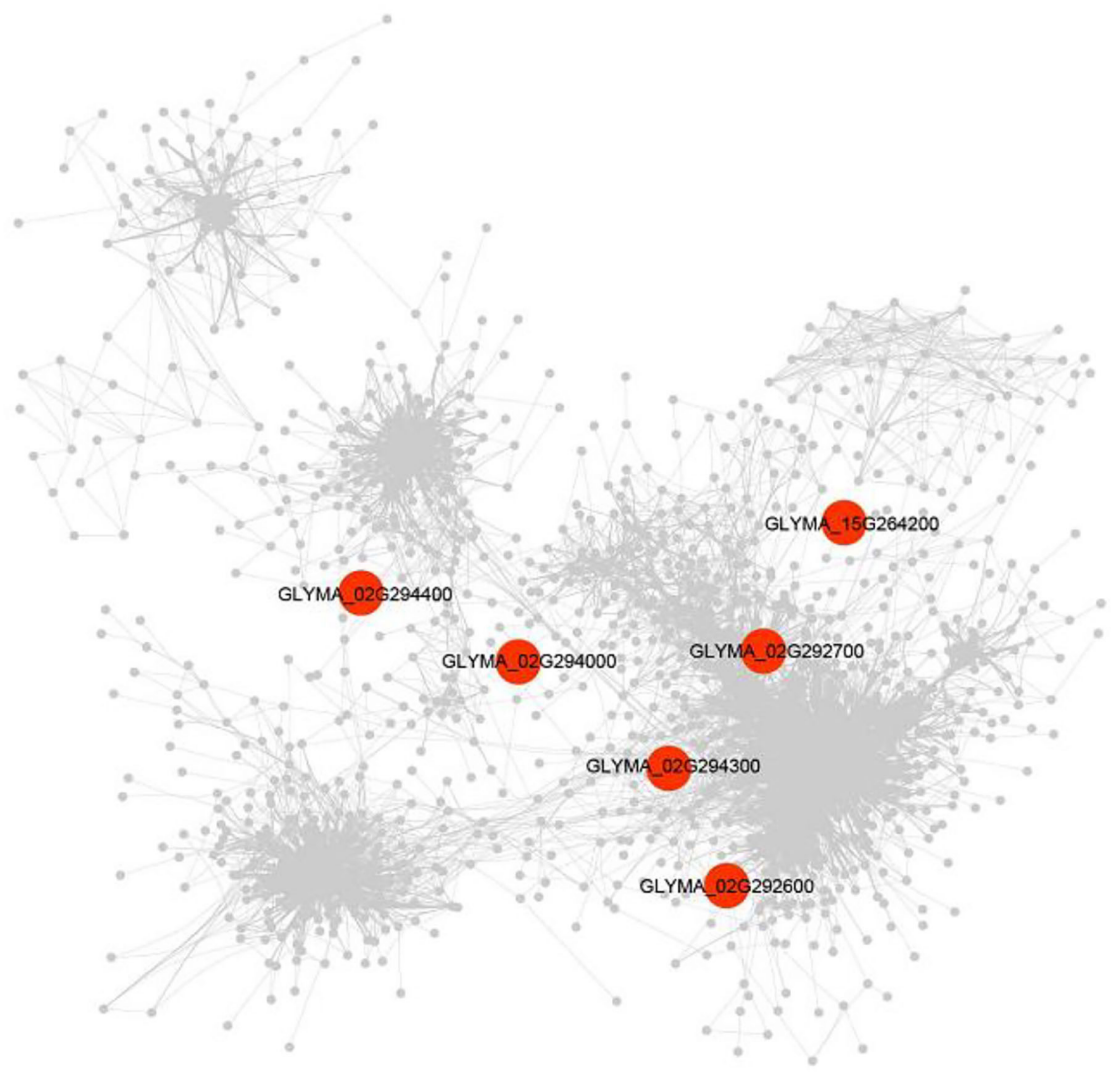
A gene co-expression network underlying soluble sugar content. The co-expression was performed with transcriptome data under high- vs. low-soluble sugar content (*n* = 1,515). Red shapes represent the genes that were detected by GWAS.

The expression of the *Glyma.02G292600* gene was identified to be correlated with 15 genes, involving integrase-type DNA-binding superfamily protein (*Glyma.13G151900, r* = 0.93), glycosyl hydrolase 9B8 (*Glyma.14G019900, r* = 0.91), and oxidative stress 3 (*Glyma.05G225900, r* = 0.91). The *Glyma.02G294000* gene was found to be correlated with only one gene, which is LRR family protein (*Glyma.16G191300, r* = 0.87) (Figure 7). Meantime, *Glyma.02G294000* was found by co-expression and candidate gene-based association analysis and was used as candidate genes for soluble sugar content. In addition, *Glyma.02G292700, Glyma.02G294300, Glyma.02G294400*, and *Glyma.15G264200* genes were discovered to be correlated with 53, 4, 1, and 1 genes, respectively (Supplementary Table S6).

## Discussion

Sweetness is an important aspect of the edible quality of vegetable soybeans, and it is one of the distinguishing characteristics of vegetable soybeans from ordinary grain soybeans. The sweetness of vegetable soybeans is mainly attributed to the soluble sugar content, of which the sucrose content accounts for about 71% of the total soluble sugars, which is the key factor determining the sweetness of vegetable soybeans; also, it is highly correlated with the food quality score of vegetable soybeans. Soluble sugar is a major indicator of the intrinsic quality of vegetable soybean. How to increase the soluble sugar content of vegetable soybeans has become an urgent problem to be solved in production. The breeding of soybean lines with suitable soluble sugar content has become the main aspect of soybean fabric breeding. Studies have shown that there were abundant genetic variations in sugar components and total soluble sugar content of soybean germplasm, which could provide abundant germplasm resources for breeding (Geater and Fehr, 2000; Hou et al., 2009). For this study, 278 soybean germplasms were selected for the evaluation of soluble sugar content in fully developed seeds at three locations. In these environments, the soluble sugar content of these tested germplasms demonstrated an extensive variation and the range of variation was similar to previous studies (Hymowitz et al., 1972, 1974). In addition, the soluble sugar content varies greatly in different environments. Until now, few QTLs associated with a soluble sugar content of soybean seeds were reported by linkage analysis. Totally, thirty-seven QTLs related to sucrose concentration and fifteen QTLs related to oligosaccharide concentration were identified (http://www.soybase.org). GWAS was proven to be beneficial for the identification of candidate loci that correlated with numerous traits in crop plants such as soybean (Bandillo et al., 2015; Sonah et al., 2015; Vuong et al., 2015; Patil et al., 2016). A GWAS analysis was performed to sustain the QTL analysis and the major QTL (qSuc_08) for sucrose they identified through more than 91,000 SNPs derived from the cross of *G*. max (Williams 82) and *Glycine soja* (PI 483460B) (Patil et al., 2018). For our study, a total of thirteen QTNs related to total soluble sugar content were detected. The QTLs identified in this study did not overlap with previous studies, but some of the candidate genes near the QTLs identified in this study have been shown to be associated with soluble sugar content. Furthermore, the results of our study for QTNs detection indicate that the total soluble sugar content of tested soybean germplasms with excellent alleles was higher than soybean germplasms with other alleles. Therefore, these identified QTNs were effective for regulating the soluble sugar content in soybean.

At present, hundreds of candidate genes are involved in the metabolism process of soluble sugar in soybean (http://www.soybase.org). GWAS was the primary selection for excavating and evaluating major genes due to the relatively short LD fragments (Li et al., 2015). A total of 115 genes in the 200-kb flanking regions of the thirteen identified QTNs were considered candidate genes in this study. Among them, a total of four genes, including *Glyma.02G293900, Glyma.02G294000, Glyma.12G069000*, and *Glyma.19G072300*, were proven to be involved in the metabolism of soluble sugar content in plants. To verify the accuracy of these candidate genes, we performed gene-based association analysis and haplotype analysis. As a result, seventeen candidate genes with 84 significant SNPs were detected (Supplementary Tables S3, S4). *Glyma.02G293900* and *Glyma.02G294000* were the two genes we further validate that were associated with sugar metabolism in soybean, which indicated the reliability of our research. To further explore the candidate genes, we used 14 materials with extreme phenotypic values (seven high-soluble sugar germplasms and seven low-soluble sugar germplasms) to analyze the candidate gene expression levels. The results indicated that the expression levels of *Glyma.02G293200* and *Glyma.02G294900* were significantly positively correlated with soluble sugar content, and *Glyma.02G294000* was significantly negatively correlated with soluble sugar content. *Glyma.02G294000* is a member of O-Glycosyl hydrolases family 17 protein that is involved in the metabolic process of starch and sucrose and may affect the degradation of UDP-glucose and the content of soluble sugars. The expression levels of these three candidate genes may directly regulate the soluble sugar content in fully developed grains of soybean. In this study, we found that the *Glyma.02G294000* gene was involved in the metabolic process of starch and sucrose. The co-expression analysis found that *Glyma.02G294000* gene was found to be correlated with LRR family protein (*Glyma.16G191300, r* = 0.87). Therefore, we speculate that the *Glyma16g191300* gene may be involved in the accumulation of soluble sugar content. Whether other candidate genes may play a role in the soluble sugar content of soybean seeds is also worth exploring. Therefore, the candidate genes and beneficial alleles we detected are valuable for increasing the content of soluble sugar in soybean.

## Data Availability Statement

The original contributions presented in the study are publicly available. This data can be found here: https://www.ebi.ac.uk/eva/?eva-study=PRJEB55008. Any queries should be directed to the corresponding author.

## Author Contributions

YH designed and supervised the research and contributed to genotyping. WL and MS conceived the study and contributed to population development. XZ and HJ contributed to phenotypic evaluation. DH and XY analyzed the data. WL, MS, and YH wrote the manuscript. All authors read and approved the final manuscript.

## Funding

This study was financially supported by the National Natural Science Foundation of China (31971967 and 31871650), the National Key Research and Development Program of China (2021YFD1201604 and 2019YFD1002601), the Youth and Middle-aged Scientific and Technological Innovation Leading Talents Program of the Crops (2015RA228), the National Ten Thousand Talent Program (W03020275), the Postdoctoral Scientific Research Development Fund of Heilongjiang Province (LBH-Z15017 and LBH-Q17015), and the Program on Industrial Technology System of National Soybean (CARS-04-PS06).

## Conflict of Interest

The authors declare that the research was conducted in the absence of any commercial or financial relationships that could be construed as a potential conflict of interest.

## Publisher's Note

All claims expressed in this article are solely those of the authors and do not necessarily represent those of their affiliated organizations, or those of the publisher, the editors and the reviewers. Any product that may be evaluated in this article, or claim that may be made by its manufacturer, is not guaranteed or endorsed by the publisher.

## Supplementary Material

Supplementary Figure S1 Functional categories of the candidate genes related to soluble sugar content in soybean.

Supplementary Figure S2 Soluble sugar content of 14 soybean germplasms. 1–7 represent germplasm with low-soluble sugar content, and 8–14 represent germplasm with high-soluble sugar content.

Supplementary Table S1 Source of 278 soybean accessions.

Supplementary Table S2 PCR primers used for expression analysis by RT-qPCR.

Supplementary Table S3 Haplotype analysis of candidate genes.

Supplementary Table S4 Haplotypes analysis of genes related to total soluble sugar content. Significance of ANOVA: ^*^*P* < 0.05, ^**^*P* < 0.01.

Supplementary Table S5 Correlation analysis of soluble sugar contents with expression levels of candidate genes.

Supplementary Table S6 The Pearson correlation analysis of transcriptome data.
