# Supplementary figures and images for: Genome-Wide Identification of Candidate Genes Underlying Soluble Sugar Content in Vegetable Soybean (Glycine max L.) via Association and Expression Analysis
Source: Front Plant Sci. 2022 Aug 4;13:930639. doi: 10.3389/fpls.2022.930639 (PMC9387354; doi:10.3389/fpls.2022.930639)

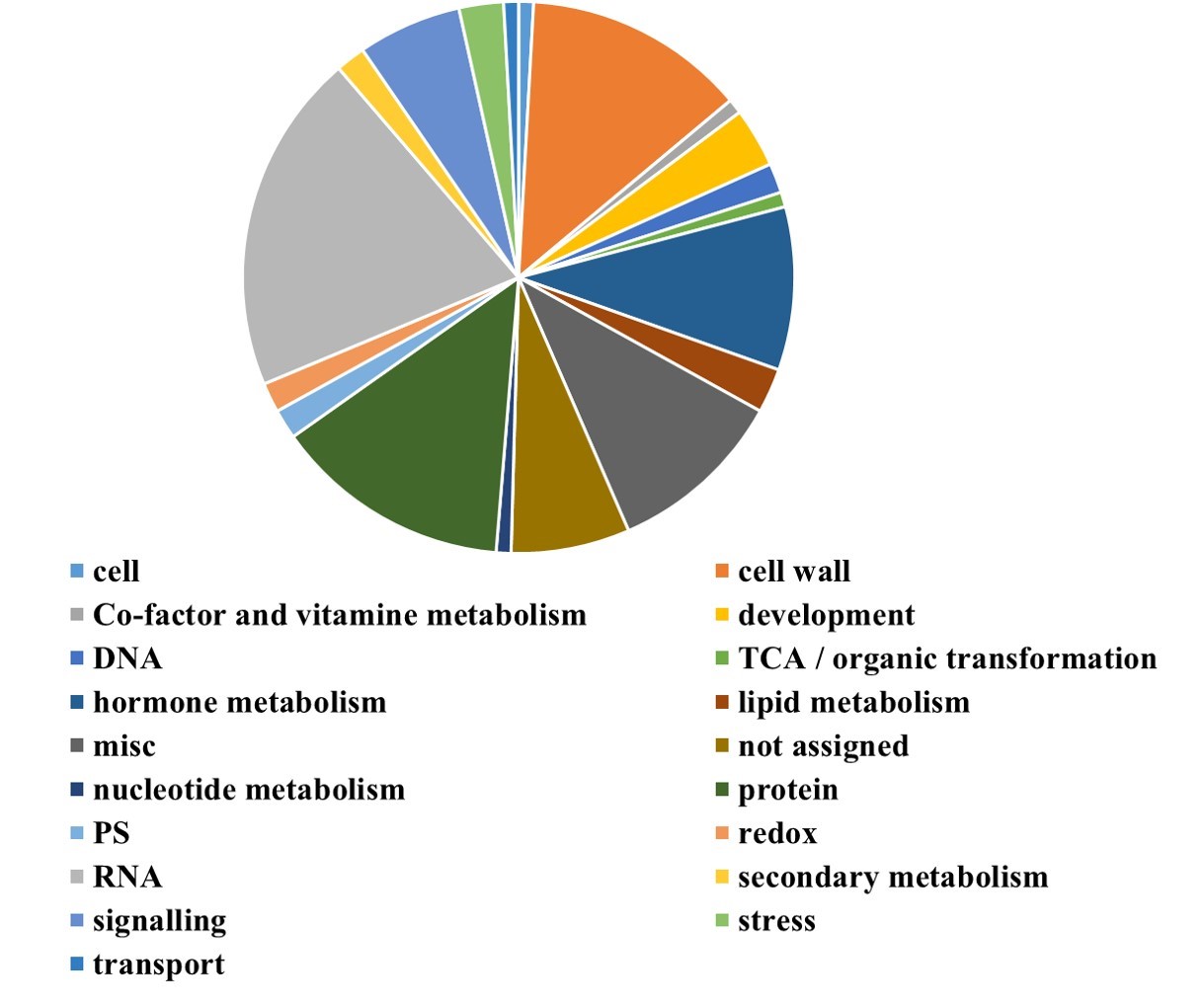

Supplement: Supplementary Figure S1 — Functional categories of the candidate genes related to soluble sugar content in soybean. [file Data_Sheet_1.ZIP › Supplementary material/Figure S1.jpg]

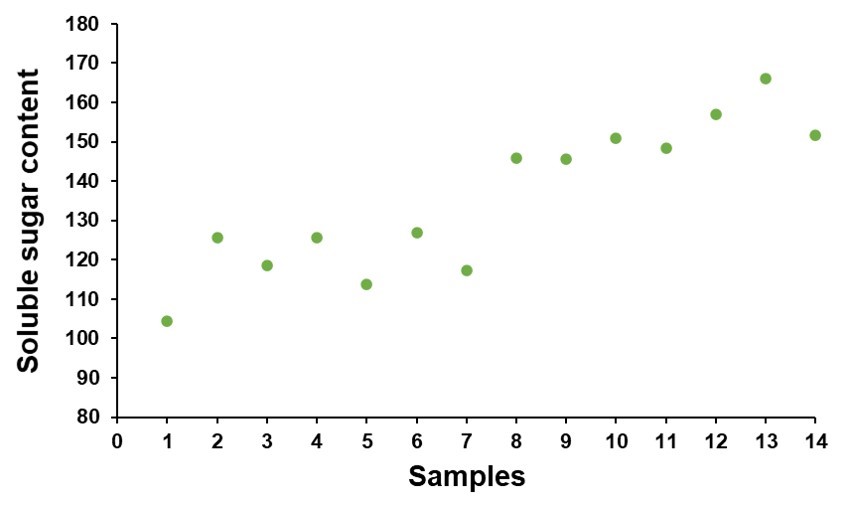

Supplement: Supplementary Figure S1 — Functional categories of the candidate genes related to soluble sugar content in soybean. [file Data_Sheet_1.ZIP › Supplementary material/Figure S2.jpg]
